# Comparing Antegrade and Retrograde Parotidectomy: Surgical Parameters and Complications

**DOI:** 10.22038/IJORL.2022.51069.2717

**Published:** 2022-03

**Authors:** Kamran Khazaeni, Bashir Rasoulian, Elahe Sadramanesh, Leila Vazifeh Mostaan, Leila Mashhadi, Golnaz Gholami

**Affiliations:** 1 *Department of Otorhinolaryngology Head and Neck Surgery, Mashhad University of Medical Sciences, Mashhad, Iran.*; 2 *Sinus and Surgical Endoscopic Research Center, Mashhad University of Medical Sciences, Mashhad, Iran.*; 3 *Otorhinolaryngologist* *, Mashhad,* *Iran.*; 4 *Department of Anesthesiology, Mashhad University of Medical Sciences, Mashhad, Iran* *.*; 5 *Nuclear Medicine Physician, Mashhad University of Medical Sciences, Mashhad, Iran.*

**Keywords:** Aantegrade dissection, Facial Nerve, Parotid Mass, Retrograde dissection

## Abstract

**Introduction:**

Patotidectomy is the treatment of choice for superficial parotid gland lesions. The present study aimed to assess the facial nerve status, as well as peri-and postsurgical complications, in two surgical techniques (antegrade and retrograde) for parotidectomy.

**Materials and Methods:**

This study was conducted on 56 patients diagnosed with parotid neoplasms from 2013-2015. The patients were randomly assigned to two groups of antegrade and retrograde. In the retrograde group, the dissection was performed initially to expose the facial nerve branches, while in the antegrade approach, the facial nerve trunk was exposed initially. Different values, such as intraoperative bleeding, mass characteristics, and the time for different sections of the surgery, were noted. The facial nerve was examined after the surgery; moreover, hospital stay and drain removal time was also noted. During the six-month postoperative period, complications and squeals were also noted.

**Results:**

Based on the results, antegrade nerve dissection was performed in 24 patients, while retrograde nerve dissection was carried out in 25 patients. The two groups were compared for intraoperative bleeding, drain output, and drain removal time. Hospital stay was found to be statistically higher in the retrograde group (P<0.05). Other complications and morbidities, such as facial nerve trauma, sialoceles, salivary fistulas, Frey’s syndrome, skin sensory changes, and surgery time, were not statistically different (P≥0.05).

**Conclusions:**

As evidenced by the obtained results, retrograde dissection had higher intraoperative bleeding and longer hospital stay. It seems that skin flap dissection is more extensive in retrograde dissection, leading to more bleeding in this approach. These differences, although statistically significant, are not clinically important; consequently, surgeons’ experience and knowledge about the two approaches are of utmost importance.

## Introduction

Salivary gland tumors account for 3%-6% of the head and neck neoplasms, 80% of these tumors occur in the parotid gland, and the majority (80%) of them are benign (1). Parotidectomy is the treatment of choice for superficial parotid gland lesions. Since the distal branches of the facial nerve are in close contact with the parotid tissue, identification and preservation of the facial nerve are among the major contributors to success in parotid surgery (2,3). In case of intact facial nerve function before surgery, apart from preserving the functional integrity, the surgeon must control the existing pathological condition (either benign or malignant) (4). The anatomy of the facial nerve after emerging from the stylomastoid foramen has different variations; nonetheless, the most common morphology of the facial nerve has been reported in the available literature (5-7). 

The standard and traditional method for parotidectomy is the antegrade approach toward the facial nerve. In this technique, the nerve trunk is identified at the point emerging from the stylomastoid foramen. The landmarks used for identifying the nerve trunk are the tympanomastoid suture, the tragal pointer, and the posterior belly of the digastric muscle (8). The retrograde approach includes approaching the proximal segment of the parotid gland via the identification of the distal branches of the facial nerve. Considering that the facial nerve trunk in certain cases, such as obesity, large tumors, or revision surgery, can be quite challenging even for well-experienced surgeons, the application of retrograde approach and familiarity of surgeons with this technique is highly recommended (9). 

The present study aimed to assess the facial nerve status in the two mentioned surgical techniques; moreover, the peri- and postsurgical complications were compared in these patients.

## Materials and Methods

This clinical trial was conducted on all patients diagnosed with parotid neoplasm who underwent surgery in the Otorhinolaryngology clinic of Qaem Hospital, Mashhad, Iran, from 2013-2015. Patients with a history of previous surgery, underlying coagulopathy state, and hemorrhagic disorders were excluded from the study. Before surgery, the benefits and possible complications were described to each patient individually, and written informed consent was obtained before the surgical procedure.

The patients were then randomly assigned to two groups: 25 subjects underwent surgery by the retrograde technique, while 24 patients were operated by the antegrade approach. All of the surgeries were performed by the same experienced head and neck surgeon. Parotidectomy was performed under general anesthesia and only a single dose of muscle relaxant was used as anesthesia induction. Muscle relaxants were not repeated during the operation.

The standard Blair incision was used in all patients. The skin flap was raised in the subcutaneous plane in the pre-auricular region and the sub-platysmal plane in the neck. In the antegrade approach, which is the conventional and standard method for nerve identification and dissection, the nerve trunk was identified as it emerges from the stylomastoid foramen. In this method, the tragal pointer is used as a landmark for nerve trunk identification; the nerve trunk lies 1-1.5cm inferior and deep to the tragal pointer. When the trunk was identified, the parotid tissue was dissected anteriorly up to the first nerve branch, the other branches were traced by the same method, and the mass, along with parotid tissue, was resected lateral to the facial nerve branches. 

In the retrograde approach, the subcutaneous flap was elevated beyond the anterior parotid border. The peripheral nerve branches were normally identified by dissection at the anterior border of the parotid gland on the masseter muscle, and the nerve trunk was exposed in a retrograde fashion. Based on the position of the mass, the exposure of two or more branches of the nerve was required. In this manner, the nerve trunk was reached by dissecting a few branches, and the superficial parotid tissue and mass were resected together.

In this study, nerve monitoring was objectively performed, and in the case of facial muscle contraction, proximity to the nerve was mentioned by the surgeon's assistant. In some cases, due to mass localization, a change was required in the surgical technique during surgery, and these cases were excluded from the study. During surgery, the surgical time was recorded separately as skin incision to complete flap raise, nerve dissection initiation to the appearance of the first nerve branch or nerve trunk, first nerve branch appearance to the parotid tissue and mass resection, and finally, the completion time of the operation. The perioperative bleeding was measured by the number of blood-soaked gauzes and the blood volume in the suction device (each gauze was estimated to contain 20cc of blood). Bipolar cautery was used for bleeding control while suture ligature and the harmonic device were not applied. At the end of the operation, the hemovac drain was inserted and the post-surgical blood loss was measured based on the amount of blood inside the drain on a daily basis. When the drainage volume was less than 20cc in two consecutive days, the drain was removed and the removal time was recorded. The patient was discharged on the same day and the length of hospitalization was also recorded. During the six postsurgical months, the patients were examined and studied for complications, such as sialocele, salivary gland fistula, facial nerve function, ear lobule paresthesia, skin discoloration, apparent skin depression, and Frey syndrome. 

## Results

A total of 56 patients who were candidates for parotidectomy due to a parotid mass were included in this study. Seven patients were excluded due to a change in the surgical technique during the operation. The patients’ mean age was reported as 42.6±15.4 years (ranging from 15-78 years). 

In total, 25 patients were treated with the retrograde technique, and 24 cases underwent the antegrade approach. In the histological examination of parotid masses, 45 (91.8%) cases were reported as benign and 4 (8.1%) subjects were malignant. The most prevalent benign tumor was pleomorphic adenoma (n=35), whereas the most common malignant tumor was mucoepidermoid carcinoma (n=3). The comparative results on perioperative bleeding volume, postoperative drainage volume, postoperative drainage removal time, and length of hospitalization are presented in Table 1. Moreover, peri- and post-operative complications are displayed in Table 2.

**Table 1:** Comparative results on surgical time, perioperative bleeding volume, postoperative drainage volume, postoperative drainage removal time, and hospitalization duration between two techniques (antegrade vs retrograde)

**Variables**		**Ante grade group**	**Retrograde group**	**P-value** **(Mann-Whitney)**
surgical Times: (min)	Time Interval from the beginning of surgery to the appearance of the first nerve branch/nerve trunk	42.79±14.7	39.36±13.8	0.227
	Time Interval from detection of first nerve branch/nerve trunk to the parotid mass resection	71.25±19.6	79.32±19.7	0.462
	Total length of surgery	130.21±21.24	132.0±30.0	0.585
Blood loss :(cc)	Perioperative bleeding	117.5±64.7	247.2±86.4	0.01
	Drain volume (the first day post op )	14.04±11.8	17.80±9.02	0.066
	Drain volume (the second day post op )	10.79±8.42	14.00±8.66	0.072
	Total post op drain volume	25.25±21.16	37.36±28.43	0.048
Drain removed time (day)		2.04±0.2	2.40±0.7	0.023
Length of hospitalization(day)		2.04±0.2	2.28±0.5	0.048

**Table 2:** Comparative results on peri- and postoperative complications between the two techniques (antegrade vs retrograde)

	**Retrograde**	**Anterograde**	**Total number**	**P-value**
Facial paralysis Upper lip	1 (4)	0	1 (2)	1.00
Facial paralysis Lower lip	1 (4)	2 (8.3)	3 (6.1)	0.609
Sensory change	1 (4)	2 (8.3)	3 (6.1)	0.609
Frey syndrome	1(4)	1 (4.2)	2 (4.1)	0.10
Depressed skin	1 (4)	2 (8.3)	3 (6.1)	0.609
xerostomia	1 (4)	1 (4.2)	2 (4.1)	1.00
Pigmentation change	1 (4)	0	1(2)	1.00
sialocele	1 (4)	3 (12.5)	4 (8.2)	0.349
Sensory change in earlobe	9 (36)	8 (33.3)	17 (34.7)	0.845

## Discussion

The antegrade nerve dissection approach is accompanied by certain complications in some patients: a) in tumors located in the mandibular angle, one should pass through the mass to reach the nerve trunk(it is not a reliable approach from the oncological aspect); b) in surgeries involving both the neck and parotid gland (parotidectomy with neck dissection), antegrade parotidectomy causes a separation in the parotid and neck samples (it is not acceptable from the oncological point of view; c) in some patients (e.g. patients with deep lobe tumors, tumors on the nerve trunk, or bulky tumors) the nerve trunk site changes, and therefore, it can be accidentally damaged by the routine method; d) in patients who undergo revision parotidectomy, the parotid bed at the jaw angle has fibrosis and the nerve trunk is difficult to find; therefore, it may be easier to find and dissect the nerve in the periphery; e) theoretically, in standard parotidectomy, the nerve trunk is prone to damage, whereas in the retrograde technique, a single nerve branch is subject to section. According to the aforementioned factors, the availability of an alternative method for finding the nerve seems essential. Therefore, in this research, the retrograde approach was studied and compared with the antegrade technique in different aspects. Regarding the surgical time, no significant difference was observed between the two methods (it was 130 min in the antegrade and 132 min in the retrograde technique). In a study by Shrestha et al. in 2011, these values were reported as 110 and 80 min in the two mentioned techniques, respectively, signifying lesser time in the retrograde method (10). In the same context, in a study by Emodi et al. in 2010, a remarkable difference was observed in the operation time, being less in the antegrade method (11). In theory, we expected the operative time to be less in the retrograde technique, especially when the tumor is located on the parotid tail, with no need to expose all the nerve branches or the nerve trunk. In such cases, the tumor can be resected by the retrograde technique via finding only one or two branches of the nerve. The other issue is the surgeon's experience in performing the two techniques, and in the present study, the surgeon had the same experience in this respect.

The rates of transient nerve paralysis were obtained at 8% and 8.3% in the retrograde and antegrade approaches, respectively. Paralysis of the buccal branch of the facial nerve was observed in only one case (4%) in the retrograde technique. Marginal branch paralysis occurred in 3 cases among which 1 (4%) was related to the retrograde group and 2 others (8.3%) were from the antegrade group. The mentioned buccal branch paralysis occurred in a patient with a bulky tumor in which the nerve had passed through the tumor and all around the nerve was surrounded by tumoral tissue. Therefore, we had to resect the nerve branch and nerve grafting was performed using the greater auricular nerve. In the study by Anjum et al. on 89 patients, 40 and 49 cases were operated with the retrograde antegrade approaches, respectively. Facial nerve palsy was reported as 45% in both methods, and it was temporary and not significantly different between the two groups (12). In another study by Shrestha et al., facial nerve paresis was obtained at 13% and 16% in the antegrade and retrograde techniques, respectively. Although the rate was higher in the retrograde method, it was not statistically significant (10). Regarding facial nerve paralysis, it was hypothesized that in the retrograde method, nerve branch paralysis is less probable, and in case of occurrence, it would involve one or two branches only, whereas in the antegrade method, the nerve trunk is injured, causing damage to all nerve branches. 

In the present study, the quantities of bleeding in the antegrade and retrograde methods were 117.5cc and 247.2cc, respectively, indicating a significant difference (P=0.010). The bleeding volume was measured by the number of blood-soaked gauzes (20cc for each gauze). The branches of the posterior auricular artery are encountered while using the antegrade approach which has the risk of perioperative injury and bleeding. Nonetheless, in the retrograde technique, since a larger flap is elevated with a greater risk of bleeding on that surface, the higher amount of bleeding can be justified. In a study by Tam et al., perioperative bleeding values were reported as 48.2cc and 65.8cc in the retrograde and antegrade techniques, respectively (13). This difference can be ascribed to the use of hemostasis tools during the operation. In the present study, only bipolar cautery was applied and its use was also limited due to the risk of facial nerve injury.

In the present study, the total drainage quantities were 37.36cc and 25.25 cc in the retrograde and antegrade methods, respectively (P=0.048). The increase in drainage volume in the retrograde technique can be justified by the amount of bleeding from the flap surface area. The drain was removed after 2.40 and 2.04 days in the aforementioned techniques, respectively (P=0.023). 

Therefore, the length of hospitalization which correlated with the drainage time was significantly longer in the retrograde technique. In the current study, the hospitalization time was longer due to the fact that the drain was removed later in the retrograde technique. In the histological examination of the parotid masses, 45 (91.8%) and 4 (8.1%) cases were reported as benign and malignant, respectively. All of the benign lesions underwent superficial parotidectomy. Due to the equal distribution of malignant cases in the two groups (two cases in each group), the impact of their presence (total parotidectomy) is statistically negligible. On the other hand, in the current study, the quantities of resected tissue were 26.19 and 31.32cc; moreover, its weight was 53.02 and 49.88 gr in the antegrade and retrograde techniques, respectively, indicating no significant difference between the two groups. Therefore, differences in lesion volume and extent in the two groups can not affect the other results, such as perioperative bleeding. 

In a study by Chow et al., the total quantities of resected tissue for the pathological study were 34cc and 13.9cc in the aforementioned techniques, indicating less tissue resection by the retrograde technique (13). Given that partial parotidectomy is more likely in the retrograde approach, the resected tissue was expected to be less, compared to that in the antegrade technique; nonetheless, this was not approved in the present study. Furthermore, parotidectomy-related complications, including hematoma, salivary gland fistula, wound infection, and hypertrophic scar, were observed in none of our patients. In the study by Anjum et al., the rate of wound infection rates were 12% and 8% in the retrograde and antegrade techniques, respectively. Accordingly, hematoma occurred in 12% and 6%, a hypertrophic scar in 5% and 4%, and salivary gland fistula in 2.5% and 6% in the retrograde and antegrade methods, respectively (12). In the present study, sialocele occurred in 1 (4%) and 3 (12.5%) cases, respectively; however, despite the higher rate in the antegrade method, the difference was not statistically significant.

The sialocele formed in the patient by the retrograde technique was 5cc in volume and was treated with a single aspiration. 

All the other cases of sialocele formation in the patients treated with the antegrade technique were recovered with several aspirations and compression dressing without any complications. Anjum et al. also reported sialocele formation only in 4% of the cases in the antegrade method (12). Considering that the probability of partial parotidectomy and residual parotid tissue is higher in the retrograde technique, sialocele formation was expected to be more prevalent; nonetheless, it was not confirmed by the study results. In the present study, Frey syndrome was detected in two patients (one in each group). We had hypothesized that Frey syndrome was less likely to occur in the retrograde technique due to the higher probability of partial parotidectomy and the more tissue placed under the flap. Nevertheless, no such difference was observed in this study.

## Conclusion

In general, a difference was observed between the two techniques in the amount of perioperative and postoperative bleeding, the drain removal time, and hospitalization length. Therefore, it can be concluded that certain factors affected the increased peri- and postoperative bleeding and hospitalization length in the retrograde approach, whereas larger flap dissection in the subcutaneous plane is the only different variable between the two groups affecting the bleeding volume. This difference can be neglected since it does not cause any major complications for the patient; moreover, other complications, such as facial nerve palsy, sialocele formation, salivary gland fistula, and Frey syndrome, did not differ significantly between the two groups. Therefore, the retrograde technique can be used as an alternative approach to superficial parotidectomy. Nevertheless, in parotidectomy, the surgeon's skill and experience in various methods of nerve exposure are of utmost importance, resulting in minimal risk of nerve injury.
